# Bacteriostatic Mechanism of the Ethyl Acetate Extract from the Root of *Schisandra propinqua* (Wall.) Baill. var. *sinensis* Oliv (Xiao Xue Teng) Against *Staphylococcus aureus*

**DOI:** 10.3390/vetsci13030285

**Published:** 2026-03-18

**Authors:** Lingyun Gu, Huifang Zhou, Qunxin Wang, Weidong Sun, Fuxin Chen, Tuo Li, Chenghua He

**Affiliations:** 1 College of Veterinary Medicine, Nanjing Agricultural University, Nanjing 210095, China; gulingyun@stu.njau.edu.cn (L.G.); 2024107089@stu.njau.edu.cn (H.Z.); 2024807144@stu.njau.edu.cn (Q.W.); swd100@njau.edu.cn (W.S.); 2 School of Chemistry and Chemical Engineering, Xi’an University of Science and Technology, Xi’an 710054, China; chenfuxin@xust.edu.cn; 3 Dalian Institute of Marine Traditional Chinese Medicine, Dalian University, Dalian 116622, China; lituo@dlu.edu.cn

**Keywords:** *Staphylococcus aureus* ATCC 25923 (*S. aureus* ATCC 25923), the ethyl acetate extract from the root of *Schisandra propinqua* (Wall.) Baill. var. *sinensis* Oliv (Xiao Xue Teng), bacteriostatic mechanism, oxidative stress

## Abstract

Background: The root of *Schisandra propinqua* (Wall.) Baill. var. *sinensis* Oliv is a traditional ethnomedicine in China; it was widely used to treat abscesses, sores, carbuncles, rheumatism, and so on. The purpose of this study was to elucidate the bacteriostatic mechanism of the ethyl acetate extract from the root of *Schisandra propinqua* (Wall.) Baill. var. *Sinensis* Oliv (Xiao Xue Teng) against *Staphylococcus aureus* ATCC 25923 (*S. aureus* ATCC 25923). Methods: Bioactive bacteriostatic constituents in Xiao Xue Teng were identified through Hybrid Quadrupole-TOF LC/MS/MS. The minimum inhibitory concentration (MIC) of Xiao Xue Teng against *S. aureus* ATCC 25923 was determined using the microbroth dilution method. A time–kill curve analysis was used to evaluate the bacteriostatic effects. SDS-PAGE coupled with nano-liquid NanoLC-ESI-MS/MS, real-time PCR, and scanning electron microscopy (SEM) was used to study the bacteriostatic mechanism of Xiao Xue Teng against *S. aureus* ATCC 25923. Results: The MIC of Xiao Xue Teng against *S. aureus* ATCC 25923 was determined to be 15.625 µg/mL. The translation initiation factor (IF-2) and elongation factor (EF-Tu) were significantly decreased in *S. aureus* ATCC 25923 after treatment with Xiao Xue Teng, while the proteins SodA and AhpC were obviously increased. The intracellular levels of total reactive oxygen species (ROS) and hydrogen peroxide (H_2_O_2_) were significantly increased (*p* < 0.01) after the treatment with Xiao Xue Teng. Concurrently, the activities of SOD, CAT and GSH-Px were significantly increased (*p* < 0.01). Moreover, cellular swelling and shrinkage were observed using SEM. Conclusions: The bacteriostatic mechanism of Xiao Xue Teng against *S. aureus* ATCC 25923 was related to eliciting oxidative stress, inhibiting protein synthesis and enhancing cytoplasmic membrane permeability.

## Simple Summary

*Staphylococcus aureus* (*S. aureus*) is an important pathogen of zoonoses including skin abscesses, pneumonia and sepsis in humans and dermatitis, mastitis, abscesses and arthritis in animals. However, the clinical treatment of staphylococcosis has become increasingly difficult due to the overuse of antibiotics, which has led to the development of antibiotic resistance. Consequently, developing safe antimicrobial strategies to reduce antibiotic resistance has become imperative. *Schisandra propinqua* (Wall.) Baill. var. *sinensis* Oliv, a member of the magnoliaceae family, was documented in the Chinese Materia Medica. The root of *Schisandra propinqua* (Wall.) Baill. var. *sinensis* Oliv has been used to treat trauma-related injuries, abscesses, carbuncles, and so on. However, the bacteriostatic mechanism against *S. aureus* in *Schisandra propinqua* (Wall.) Baill. var. *sinensis* Oliv has never been reported. This study investigated the bacteriostatic mechanism of the ethyl acetate extract from the roots of *Schisandra propinqua* (Wall.) Baill. var. *sinensis* Oliv (Xiao Xue Teng) against *S. aureus* ATCC 25923. The results showed that Xiao Xue Teng exerted a bacteriostatic effect against *Staphylococcus aureus* ATCC 25923 by eliciting oxidative stress, disturbing protein synthesis and enhancing cytoplasmic membrane permeability.

## 1. Introduction

*Staphylococcus aureus* (*S. aureus*) is a clinically significant Gram-positive pathogen. Its escalating multi-drug resistance (MDR) poses substantial threats to global public health and animal welfare [1]. In human health, *S. aureus* has been mainly responsible for community and hospital infections (such as skin abscesses, pneumonia and sepsis) and was one of the leading causes of bacterial food poisoning worldwide [2]. Previous studies showed that *S. aureus* prevalence differs significantly among various host species. Approximately 30% to 80% of humans were carriers of *S. aureus*, whereas carriage rates in domestic animals could be as high as 90% in chickens, 77% in dairy cows and heifers, 43% in pigs, and 29% in sheep [3]. Moreover, the isolation of *S. aureus* from a wide range of wildlife specimens further confirmed its broad ecological distribution [4]. *S. aureus* infection can cause dermatitis, mastitis, abscesses and arthritis in various animals including bovine [5], chickens [6], sheep, pigs, horses, dogs and other species [7].

Notably, with the overuse of antibiotics, the treatment of *S. aureus* infection has become increasingly difficult, leading to a significant increase in mortality associated with multi-drug-resistant strains [8]. Consequently, developing safe antimicrobial strategies while reducing antibiotic resistance is imperative [9]. Botanical medicines offer distinctive advantages through their poly-pharmacological mechanisms, especially in terms of anti-bacterial activity [10].

*Schisandra propinqua* (Wall.) Baill. var. *sinensis* Oliv, a member of magnoliaceae plant, is widely distributed in the Yunnan–Guizhou Plateau of China. It is an important Miao ethnomedicine recorded in the Chinese Materia Medica. The root of *Schisandra propinqua* (Wall.) Baill. var. *sinensis* Oliv was used to treat trauma-related injuries, suppurative infections (e.g., abscesses and carbuncles), rheumatic disorders, and numbness. Phytochemical investigations have identified various compounds in *S. propinqua* var. *sinensis,* including lignans, triterpenoids, flavonoids, coumarins, iridoids, phenolic acids and polysaccharides. These chemicals have multifaceted pharmacological effects including antioxidant effects, anti-tumor properties, and gut microbiota-modulated immunoenhancement [11,12].

Previous studies have demonstrated that crude extracts and purified compounds from Schisandra species exhibited antibacterial activity against a range of pathogenic bacteria. A summary of the reported antibacterial effects of Schisandra-derived substances is presented in Table 1.

Nevertheless, the bacteriostatic mechanism against *S. aureus* in *Schisandra propinqua* (Wall.) Baill. var. *sinensis* Oliv has never been reported. This study therefore aimed to elucidate the bacteriostatic mechanism of the ethyl acetate extract from the root of *Schisandra propinqua* (Wall.) Baill. var. *sinensis* Oliv against *S. aureus* ATCC 25923.

## 2. Materials and Methods

### 2.1. Bacterial Strain and Culture Conditions

The bacterial strains *S. aureus* ATCC 25923, MRSA ATCC 43300 and clinical isolate JS-25-01 from a poultry farm in Jiangsu province, previously preserved in the Clinical Microbiology Laboratory at Nanjing Agricultural University, were employed in this study.

Primary cultures were initiated by inoculating *S. aureus* ATCC 25923, MRSA ATCC 43300 and clinical isolate JS-25-01 into Mueller Hinton broth (MHB, Qingdao Hope Bio-Technology Co., Ltd., Qingdao, China) followed by overnight incubation at 37 °C and at 180 rpm, respectively. Subsequently, the revived cultures were streaked onto mannitol salt agar plates using a sterile loop. All plates were inverted and maintained at 37 °C for 12 h in a constant temperature incubator. After that, the characteristic yellow colony of *S. aureus* was selected and streaked a second time to ensure strain purity. The isolated colony from the second plate was inoculated into MHB for 8 h, and then the working bacterial suspension for the experiment was yielded.

### 2.2. Preparation of the Ethyl Acetate Extract

In the autumn of 2023, the root of *Schisandra propinqua* (Wall.) Baill. var. *sinensis* Oliv was collected from Longping Town in Guizhou Province and identified by Dr. Fan Fujian and was then deposited (Herbarium no. 20230810) at the herbarium of the College of Veterinary Medicine, Nanjing Agricultural University (NJAU), Nanjing, China.

Dried roots of *Schisandra propinqua* (Wall.) Baill. var. *sinensis* Oliv (200 g) were fragmented using a mechanical grinder and extracted with 600 mL ethyl acetate in an Erlenmeyer with ultrasonic-assisted extraction (40 min). After 24 h maceration at ambient temperature (25 ± 2 °C), the filter liquor was collected through a Buchner funnel connected to a vacuum pump. The collected filtrate was concentrated under reduced pressure at 40 °C using a rotary evaporator (RE-3000A, Shanghai Yarong Biochemistry Instrument Factory, Shanghai, China), and then dried at 50 °C in a oven [22]. The yield of extract was 8.9% (*w*/*w*). The extract was stored in a refrigerator at 4 °C for future use. The ethyl acetate extract from the roots of *Schisandra propinqua* (Wall.) Baill. var. *sinensis* Oliv was abbreviated as Xiao Xue Teng.

### 2.3. HPLC-HRMS Assay

Xiao Xue Teng was detected using high-performance liquid chromatography (HPLC) equipped with a UV detector and autosampler (Shimadzu, Kyoto, Japan). The analytical column was Agilent TC-C18 column (250 mm × 4.6 mm, 5 μm, Agilent, Santa Clara, CA, USA). Mobile phases: A: 95% acetonitrile + 0.1% formic acid; B: 5% acetonitrile + 0.1% formic acid. Gradient program: 0.01–1 min: 40% B; 1–11 min: 80% B; 11–26 min: 100% B; 26–30 min: 60% B. Detection: 254 nm. Flow rate: 0.4 mL/min. Column temperature: 35 °C. Injection volume: 10 μL. High-resolution mass spectrometry (HRMS) analysis was performed using a Hybrid Quadrupole-TOF LC/MS/MS platform equipped with a TripleTOF 4600 mass spectrometer (AB SCIEX, Framingham, MA, USA) and DuoSpray Ion Source. Mass condition included source temperature 500.0 °C, start mass 100.0 Da and end mass 1000.0 Da. Data were analyzed using the software PeakView with XIC manager (version 1.2.0.3, AB SCIEX, Framingham, MA, USA).

### 2.4. Microbroth Dilution MIC Assay

The microdilution technique was used to determine the MIC of Xiao Xue Teng in the 96-well plates. Briefly, 100 μL of Xiao Xue Teng (final concentration 2 mg/mL) was double diluted into the 96-well plate from column 1 to column 10. Column 11 contained ceftiofur sodium (final concentration: 0.5 μg/mL). The 96-well plate was dried at 40 °C. Then, 100 μL of *S. aureus* ATCC 25923, MRSA ATCC 43300 and clinical isolate JS-25-01 suspension (1 × 10^6^ CFU/mL) was added, respectively. Two duplicate experiments were conducted for each strain. The plate was incubated in incubator at 37 °C for 12 h. Finally, 50 uL of resazurin (0.5 mg/mL, Sigma-Aldrich, Shanghai, China) was added into each well as an indicator. After 1 h incubation at 37 °C, the lowest concentration of Xiao Xue Teng which had no color change was the MIC [23].

The MICs of Xiao Xue Teng against *S. aureus* ATCC 25923, MRSA ATCC 43300 and clinical isolate JS-25-01 were 15.625, 125 and 250 µg/mL, respectively. The strain *S. aureus* ATCC 25923 was selected to study in this paper.

### 2.5. Determination of Time–Kill Curves

Xiao Xue Teng was dissolved into tubes with MHB to achieve final concentrations of 1/4 × MIC, 1/2 × MIC, 1 × MIC, 2 × MIC and 4 × MIC. Ceftiofur sodium (final concentration, 0.5 µg/mL) served as the positive control, while only MHB was the negative control (NC). *S. aureus* ATCC 25923 was dispensed into each tube (final concentration, 1 × 10^6^ CFU/mL). All tubes were incubated at 37 °C with continuous shaking (180 rpm). Bacterial viability was assessed through the colony counting method on MH agar plates at 2 h, 4 h, 6 h, 8 h, 10 h, 12 h and 24 h, respectively [24]. The time–kill curves were generated from colony-forming unit (CFU) counts.

### 2.6. SDS-PAGE

Xiao Xue Teng was dissolved in MHB at 1 × MIC, while only MHB was the NC group. Mid-logarithmic phase *S. aureus* ATCC 25923 (final concentration, 1 × 10^6^ CFU/mL) was added into tubes and incubated at 37 °C and at 180 rpm for 12 h. Bacteria were collected through centrifugation and lysed through ice-bath ultrasonication. The protein concentration of each sample was quantified and normalized with PBS. The proteins were denatured in SDS-PAGE loading buffer and separated in a 15% SDS-PAGE gels. The gels were subsequently stained with Coomassie Brilliant Blue R-250 at 37 °C with gentle agitation for 1 h and then destained in deionized water until clear band visualization. Data were documented using an Expression 11000XL imaging system (EPSON, Nagano, Japan) and analyzed using ImageJ software (version 1.54h, available online: https://imagej.net/ij/, accessed on 15 December 2023).

### 2.7. NanoLC-ESI-MS/MS Proteomic Analysis

A sterile knife was used to excise the differentially expressed protein bands from the gel. According to the established protocols [25], the bands were digested in 100 mM ammonium bicarbonate (pH 8.5) of tryptic digestion, and the peptides were extracted by acetonitrile and completely dried under a nitrogen stream at 40 °C. A NanoLC-ESI-MS/MS system was used to analyze the peptides which dissolved in the sample solution (2% acetonitrile, 97.5% water, 0.5% formic acid). The ESI-MS/MS analysis conditions were as follow: ionization, 1.50–1.80 kV; capillary temperature, 100 °C; DDA mode, full scans (m/z 350–1650) triggering MS/MS of top 9 ions; CID energy, 33%; and dynamic exclusion, 1 repeat/1 min duration/4 Da [26]. Database searches utilized ProtQuest^®^ against UniProt with fixed carbamidomethylation and variable oxidation modifications. The data were analyzed using the software PLGS (version 2.3, Waters, Milford, MA, USA) and the KEGG database (available online: https://www.kegg.jp/).

### 2.8. The mRNA Expression Analysis of Differential Proteins

Total RNA was extracted from 1 × MIC group and the NC group using the Bacterial RNA Extraction Kit (Baisheng Biotechnology, Shanghai, China) and then reversed to cDNA with the HiScript^®^ Reverse Transcriptase Kit (Yeasen Biotechnology, Shanghai, China). The primers of *inf B*, *tuf*, *sod A*, *ahp C* and *16S* are shown in Table 2. The cDNA templates, primers and 2 × HyperMB Universal SYBR Green qPCR Master Mix (Sangon Biotech, Shanghai, China) were used in a StepOnePlus^TM^ system (Applied Biosystems, Waltham, MA, USA) with an optimized protocol: initial denaturation at 95 °C for 2 min, followed by 40 cycles of denaturation at 95 °C for 10 s and 60 °C for 30 s. Endogenous *16S* was an internal reference gene. Relative transcript quantification was performed using the 2^−ΔΔCT^ method.

### 2.9. Cytoplasmic Membrane Permeability Assessment

In this step, 1 × 10^6^ CFU/mL of *S. aureus* ATCC 25923 was incubated with 100 μM propidium iodide (PI) in PBS at 37 °C and at 180 rpm for 30 min [31]. Then, 900 μL of bacterial culture was added into 100 μL of Xiao Xue Teng (final concentration, 1/2 × MIC, 1 × MIC, 2 × MIC). An amount of 1 mL of bacteria culture with PI was set as the NC group. All groups continued to incubate in an Incubator Shaker at 37 °C and at 180 rpm for 30 min. Then, 200 μL of incubation was transferred to black 96-well plates for fluorescence measurement (λ^ex^: 535 nm, λ^em^: 615 nm) (BioTek Synergy H1, BioTek, Winooski, VT, USA).

### 2.10. Quantification of ROS

The detection process of intracellular reactive oxygen species (ROS) in bacteria was similar to the membrane permeability assessment (2.9) with only minor modifications. The fluorescence dye was changed to DCFH-DA and the detection wavelength was changed to λ^ex^: 488 nm and λ^em^: 525 nm [32].

### 2.11. Assessment of H_2_O_2_,SOD, CAT and GSH-Px in the Bacteria

Xiao Xue Teng was dissolved in MH medium at a final concentration of 1/2 × MIC, 1 × MIC and 2 × MIC. The normal MH medium was used as the NC group. Next, 1 × 10^6^ CFU/mL of *S. aureus* ATCC 25923 was respectively incubated in 1 × MIC, 2 × MIC and the NC group at 37 °C and at 180 rpm for 12 h. Then, the bacterial cells were harvested through centrifugation (4 °C, 3000 rpm, 15 min) and washed twice with 1 × PBS. Then, the bacterial cells in each group were normalized to equivalent densities using 1 × PBS and lysed through ultrasound (200 W, 15 min, 4 °C). The hydrogen peroxide (H_2_O_2_), superoxide dismutase (SOD), catalase (CAT) and glutathione peroxidase (GSH-Px) in the bacterial supernatant were quantified using commercial assay kits (Solarbio, Beijing, China).

### 2.12. Scanning Electron Microscopy (SEM)

*S. aureus* ATCC 25923 in the 1 × MIC and NC groups was collected (4 °C, 6000 rpm, 15 min), washed thrice with 1×PBS and fixed overnight in 2.5% glutaraldehyde at 4 °C. After PBS rinsing, dehydration procedures were employed using graded ethanol series (50%, 70%, 80%, 90%, 15 min each) followed by incubation with absolute ethanol for three 30 min incubations. After incubation with butanol substitution (3 × 30 min), the samples were lyophilized, sputter-coated and observed using an SEM system (Hitachi SU8010, Tokyo, Japan).

### 2.13. Statistical Analysis

All experiments were repeated three times, and data were analyzed using the software GraphPad Prism (version 9.5) with the method of One-Way ANOVA. ** means *p*-value < 0.01 and * means *p*-value < 0.05.

## 3. Results

### 3.1. HPLC and HRMS Analysis of Xiao Xue Teng

The chromatogram and total ion chromatogram (TIC) of Xiao Xue Teng detected using HPLC-HRMS are shown in Figure S1. Compared with the mass spectrum data and the literature, the chemical components in Xiao Xue Teng included Schizanrin F [33], E-Resveratrol trimethyl ether [34], Formononetin [35], arisanlactone C [36], dehydrated schizandrin [37], 7-O-Methylcedrusin [33], benzoylgomisin Q [38], Manwuwezic Acid [39], tigloylgomisin P [40], Schisantherin A [41] and alismol [42] (Table S1).

### 3.2. Time–Kill Curve

**Figure 1:**
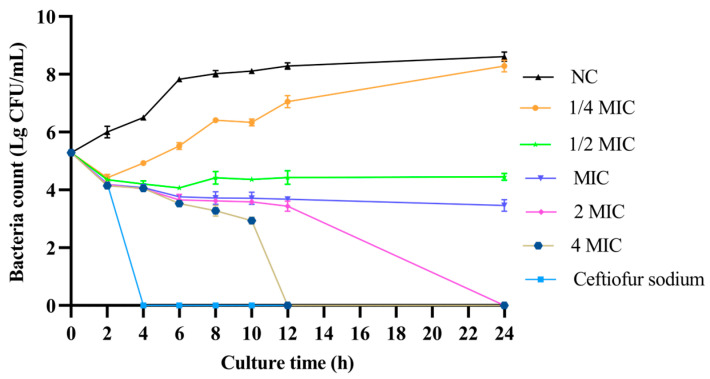
The time–kill curves for Xiao Xue Teng against *S. aureus* ATCC 25923 at different concentrations (1/4 × MIC, 1/2 × MIC, 1 × MIC, 2 × MIC and 4 × MIC). Ceftiofur sodium was used as the positive control. Normal MHB was set as the NC group.

As shown in Figure 1, when Xiao Xue Teng was used at concentration of 1 × MIC, it could inhibit the growth of *S. aureus* ATCC 25923. When the concentration of Xiao Xue Teng was increased to 2 × MIC and 4 × MIC, *S. aureus* ATCC 25923 was killed completely at 24 h and 12 h, respectively. These results demonstrated that Xiao Xue Teng exhibited bacteriostatic activity at 1 × MIC, while bactericidal effects were observed only at higher concentrations (≥2 × MIC). Xiao Xue Teng could be used as a potential multipotent bacteriostat agent against *S. aureus* infection.

### 3.3. Identification of Differentially Expressed Bacterial Proteins

**Figure 2:**
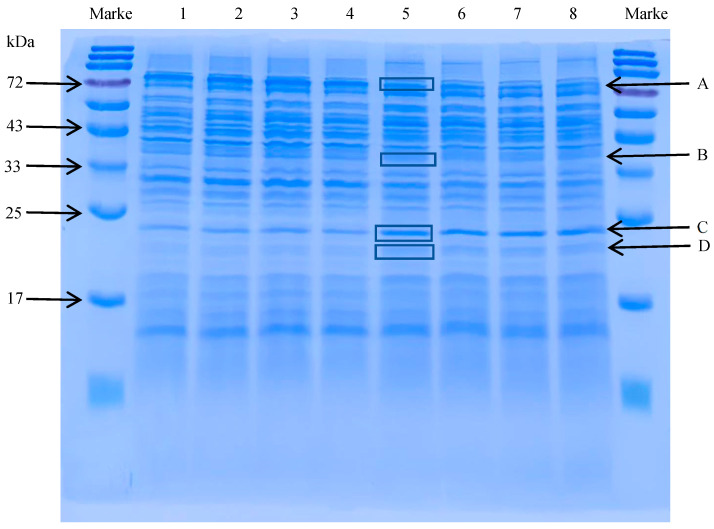
The SDS-PAGE of the total proteins from the *S. aureus* ATCC 25923 treated with Xiao Xue Teng. (Lanes 1–4: proteins from NC group; lanes 5–8: proteins from 1 × MIC group). Bands A–D denote differentially expressed proteins selected for subsequent NanoLC-ESI-MS/MS identification.

According to the results of densitometric analysis, the expressions of protein bands A (about 75 kDa) and B (35–45 kDa) were decreased, while the expressions of bands C and D (15–25 kDa) were upregulated (Figure 2). The results of NanoLC-ESI-MS/MS showed that the bands A, B, C and D were identified as factor IF-2 (encoded by gene *inf B*), elongation factor EF-Tu (encoded by gene *tuf*), Sod A (encoded by gene *sod A*) and Ahp C (encoded by gene *ahp C*), respectively. The total ion chromatograms for each identified protein band are shown in the Figure S2. Densitometric analyses of these proteins are provided in the Supplementary Materials (Figure S3). These findings demonstrated that the bacteriostatic mechanism of Xiao Xue Teng against *S. aureus* ATCC 25923 is related to oxidative stress.

### 3.4. mRNA Expression of Differential Proteins

**Figure 3:**
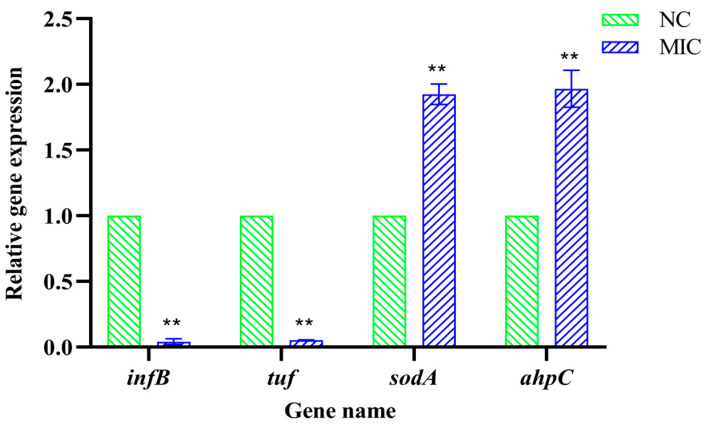
The relative gene expression of *inf B*, *tuf*, *sod A* and *ahp C* after the treatment with Xiao Xue Teng against *S. aureus* ATCC 25923. Normal MHB was used as the NC group. ** means *p*-value < 0.01.

Consistent with the protein expression data, the mRNA expressions of *inf B* (band A) and *tuf* (band B) were decreased significantly in the 1 × MIC group (*p* < 0.01). In contrast, the mRNA expressions of *sod A* (band C) and *ahp C* (band D) were increased significantly (*p* < 0.01) (Figure 3). These results showed that the expressions of RNA were consistent with the results of SDS-PAGE.

### 3.5. Cytoplasmic Membrane Permeability Alterations

**Figure 4:**
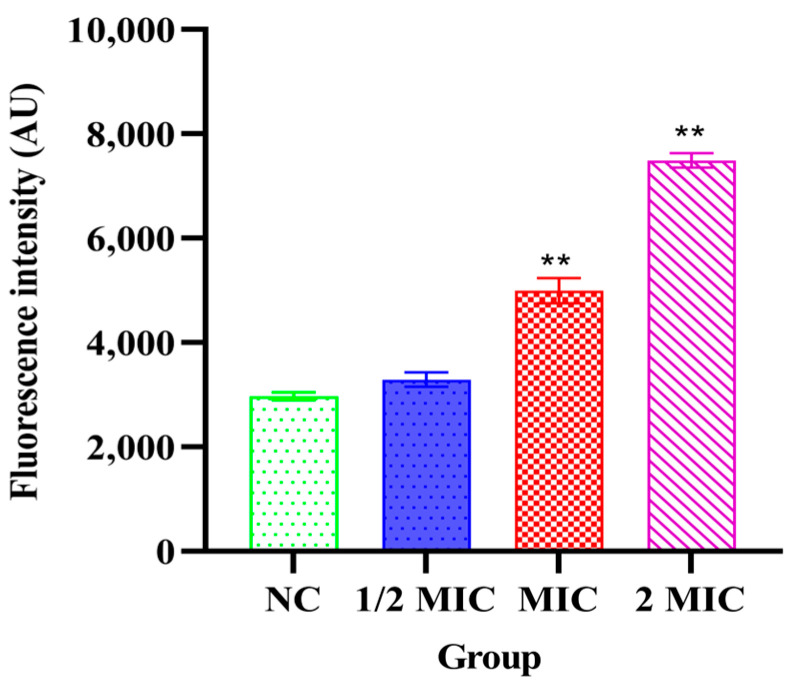
Changes in bacterial inner membrane permeability after the treatment with different concentrations of Xiao Xue Teng. Normal MHB was used as the NC group. ** indicates *p*-value < 0.01.

As shown in Figure 4, after treatment with Xiao Xue Teng at 1 × MIC and 2 × MIC, the cytoplasmic membrane permeability of *S. aureus* ATCC 25923 significantly increased compared to the NC group (*p* < 0.001). The results indicated that the cytoplasmic membrane in *S. aureus* ATCC 25923 was damaged after treatment with Xiao Xue Teng.

### 3.6. The Levels of ROS and H_2_O_2_ in the Bacteria

**Figure 5:**
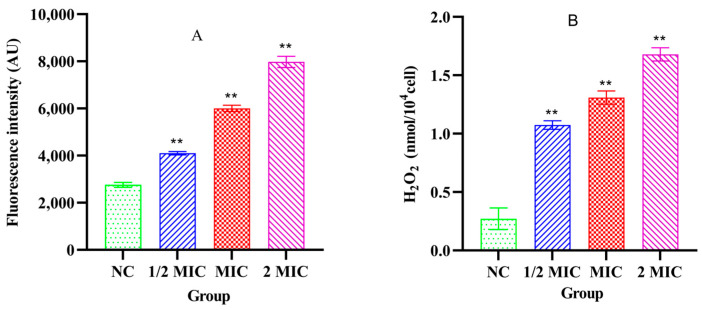
The ROS (**A**) and H_2_O_2_ (**B**) levels in *S. aureus* ATCC 25923 after the treatment with different concentrations of Xiao Xue Teng. Normal MHB was used as the NC group. ** indicates *p*-value < 0.01.

Compared with the NC group, the intracellular ROS levels in *S. aureus* ATCC 25923 were significantly increased after treatment with different concentrations of Xiao Xue Teng (1 × MIC and 2 × MIC) (*p* < 0.001). However, the intracellular ROS level in the 1/2 × MIC group was not significantly changed compared to the NC group (Figure 5A). The intracellular H_2_O_2_ levels in *S. aureus* ATCC 25923 were significantly elevated after treatment with different concentrations of Xiao Xue Teng (1/2 × MIC, 1 × MIC and 2 × MIC) (*p* < 0.001) and increased in a concentration-dependent manner (Figure 5B). These results indicated that oxidative stress in *S. aureus* ATCC 25923 was induced after treatment with Xiao Xue Teng.

### 3.7. The Levels of SOD, CAT and GSH-Px in the Bacteria

**Figure 6:**
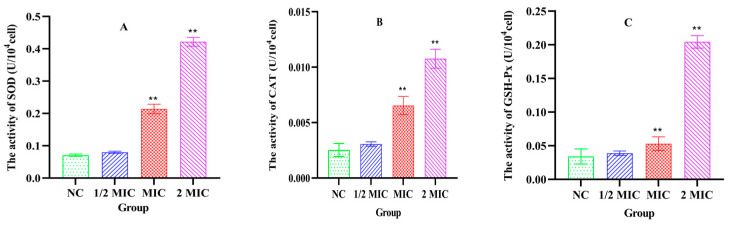
The levels of SOD, CAT and GSH-Px in *S. aureus* ATCC 25923 after the treatment with different concentrations of Xiao Xue Teng ((**A**): SOD; (**B**): CAT; (**C**): GSH-Px). Normal MHB was used as the NC group. ** indicates *p*-value < 0.01.

As shown in Figure 6, after treatment with Xiao Xue Teng (1 × MIC and 2 × MIC), the activity levels of SOD, CAT and GSH-Px in *S. aureus* ATCC 25923 were increased in comparison with the NC group (*p* < 0.001) (Figure 6). These findings showed that the antioxidant system in *S. aureus* ATCC 25923 was activated after treatment with Xiao Xue Teng.

### 3.8. Ultrastructural Morphology Analysis

**Figure 7:**
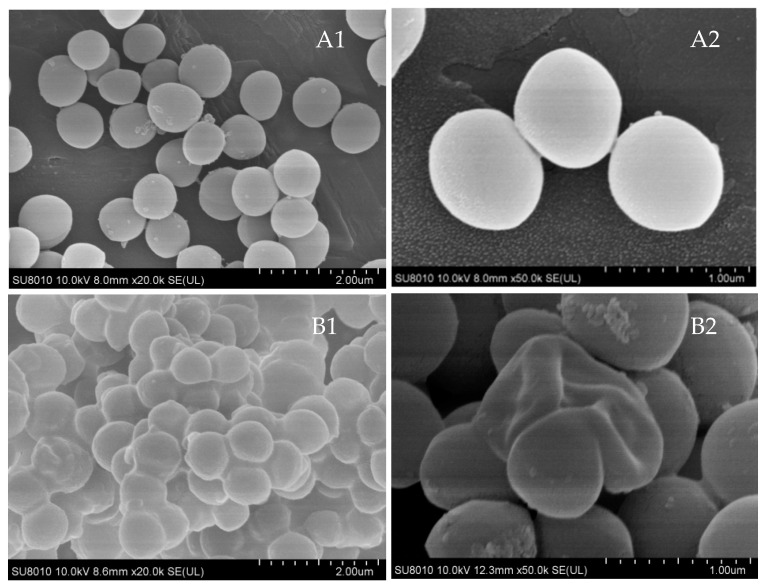
The ultrastructural changes in *S. aureus* ATCC 25923 were observed using SEM. (**A1**,**A2**) were the NC group and (**B1**,**B2**) were the 1 × MIC group ((**A1**,**B1**) 20,000×; (**A2**,**B2**) 50,000×).

In the NC group, *S. aureus* ATCC 25923 exhibited an intact cellular architecture with smooth surface topography. In contrast, the treatment with Xiao Xue Teng (1 × MIC) resulted in significant morphological alterations, including surface roughening, depression, swelling, and shrinkage (Figure 7). These observations showed that Xiao Xue Teng disrupts the membrane integrity of *S. aureus* ATCC 25923.

## 4. Discussion

The antibiotic treatment of bacterial infection has always been a problem for researchers, especially for multi-drug resistant bacteria [43]. *S. aureus* is a major pathogen responsible for skin, soft tissue, and bloodstream infections, causing significant morbidity and mortality in humans and animals worldwide [44]. After bacterial infection, especially in the later stage of infection, the treatment principle of using antibiotics is to firstly inhibit the bacterial growth and then kill them to reduce the production of endotoxin and exotoxin [45]. In this study, Xiao Xue Teng could inhibit the growth of *S. aureus* ATCC 25923 by eliciting oxidative stress, inhibiting protein synthesis and destroying the structure of bacteria. This multi-target mechanism was consistent with the characteristic of many traditional Chinese medicine extracts, which often exert antibacterial effects through the synergistic action of multiple compounds rather than a single pathway.

The main function of ribosomes in bacteria is protein synthesis by translating the genetic code of mRNA, providing all structural and functional proteins for bacterial growth, reproduction, metabolism, and stress adaptation [46]. In this study, the significant downregulation of IF-2 and EF-Tu suggested that ribosomal function was impaired following Xiao Xue Teng treatment. This finding was consistent with previous studies that demonstrated that oxidative stress could inhibit the translation process [47]. Recent studies on other natural products have similarly demonstrated that the disruption of translation factors was a key antibacterial mechanism. For instance, pseudolaric acid B from Cortex pseudolaricis has been shown to disrupt amino acid metabolism and ultimately weaken protein synthesis in fungal cells, suggesting that targeting protein synthesis machinery was a conserved strategy among natural antibacterials [48]. As a key regulator of translation initiation, IF-2 is responsible for accurately delivering fMet-tRNA to the P-site of the ribosomal small subunit. This step is essential for initiating protein synthesis. Recent studies also showed that IF-2 can influence the overall translation efficiency through its interaction with ribosome assembly factors. EF-Tu, one of the most abundant bacterial proteins, bound aminoacyl-tRNA during the elongation phase and delivered the EF-Tu-aminoacyl-tRNA complex efficiently and accurately to the ribosomal A-site. The interaction between EF-Tu and the ribosome was critical for maintaining translation speed and fidelity [49]. Moreover, H_2_O_2_ can oxidize conserved cysteine residues in EF-Tu, which could lead to a severe suppression of translation speed [50]. The downregulation of IF-2 and EF-Tu directly compromised two essential steps in protein synthesis: initiation and elongation. Given that bacteria depend on continuous protein synthesis for replication, metabolism, and stress adaptation, the impairment of these core translation factors inevitably leads to growth arrest. Based on the literature, Schisandra lignans (e.g., Schisandrin, Gomisin) contain a dihydrofuran-γ-lactone skeleton and a C-6/C-7 conjugated double bond—key structural features driving antibacterial activity [51].

The efficiency of protein synthesis determines bacterial survival under stress conditions [52]. Crucially, impaired protein synthesis can diminish the capacity to produce defense proteins in response to stress in bacteria. Thereby, the inhibition of protein synthesis could enhance the lethality induced by oxidative stress [53].

SOD and CAT are key antioxidant enzymes that protect cells against excessive ROS production [54]. The activities of SOD, CAT, and GSH-Px are increased when bacteria attempt to counteract oxidative damage. SOD converts superoxide radicals (O_2_^−^) into H_2_O_2_ and O_2_, while CAT further decomposes H_2_O_2_ into water (H_2_O) and oxygen (O_2_). This process maintains bacterial homeostasis. GSH-Px is an essential peroxidase that catalyzes the specific reduction of ROS by oxidizing reduced glutathione to its oxidized form, especially in lipid peroxidation [55]. AhpC can reduce peroxides and H_2_O_2_ to alcohols and water. The over-expression of AhpC under exogenous oxidative stress is considered protective against oxidative damage [56]. Additionally, ROS production exceeded the bacterial clearance capacity, which could lead to H_2_O_2_ accumulation. This created a vicious cycle of escalating oxidative damage, ultimately resulting in the collapse of redox homeostasis and membrane disintegration. Recent research has highlighted that targeting bacterial redox homeostasis is a promising antibacterial strategy. Studies on artemisinin derivatives and Magnolia officinalis compounds support this notion, demonstrating that disrupting bacterial redox homeostasis through ROS accumulation is an effective antibacterial strategy.

This finding may also explain the differential susceptibility observed, with Xiao Xue Teng showing an 8-fold higher MIC against MRSA compared to ATCC 25923, which is likely attributable to the enhanced oxidative stress defense systems of MRSA (e.g., upregulated SOD, CAT, and AhpC) that counteract ROS accumulation [57]. Future studies should explore combining Xiao Xue Teng with antioxidant inhibitors to synergistically enhance anti-MRSA activity, an approach supported by studies where magnolol combined with antibiotics produced synergistic effects [58].

Cytoplasmic membrane permeability is crucial for maintaining the survival of bacteria [59]. The cytoplasmic membrane is essential for bacterial survival, functioning as both a physical barrier and a hub for critical metabolic processes. Its disruption triggers bacterial death through three convergent mechanisms: the collapse of proton motive force (halting ATP synthesis and nutrient uptake), uncontrolled water influx (leading to osmotic rupture), and the dysfunction of embedded proteins (causing metabolic chaos) [60]. In Gram-positive bacteria like *S. aureus*, the absence of an outer membrane renders the cytoplasmic membrane particularly vulnerable, making its disruption rapidly lethal. The result of the cytoplasmic membrane permeability assay showed that the cytoplasmic membrane in *S. aureus* ATCC 25923 was damaged after treatment with Xiao Xue Teng. This membrane-damaging effect is a common mechanism among plant-derived antibacterials. Studies have demonstrated that various phytochemicals targeted bacterial membranes such as prenylflavonoids from Sophora flavescens, ginsenosides and polygonum chinense extract. Therefore, the primary bacteriostatic mechanisms of Xiao Xue Teng against *S. aureus* ATCC 25923 include eliciting oxidative stress, disturbing protein synthesis and enhancing cytoplasmic membrane permeability.

This study also has several limitations that should be acknowledged. The specific antibacterial compounds in Xiao Xue Teng remain unidentified due to insufficient chemical characterization, limiting reproducibility and pharmaceutical development. Future studies should not only identify active ingredients but also explore their synergistic interactions and develop quality control methods to support clinical application [37].

## 5. Conclusions

This study investigated the bacteriostatic mechanism of Xiao Xue Teng against *S. aureus* ATCC 25923 using a combination of techniques, including SDS-PAGE, real-time PCR, cytoplasmic membrane permeability assays and scanning electron microscopy. The bacteriostatic mechanism of Xiao Xue Teng against *S. aureus* ATCC 25923 mainly involves eliciting oxidative stress, disturbing protein synthesis and enhancing cytoplasmic membrane permeability. These results suggest that Xiao Xue Teng could be used as a potential multipotent bacteriostat agent against *S. aureus* infection.

## Figures and Tables

**Table 1:** Antibacterial activity of *Schisandra* spp. compounds.

Compound	Target Bacteria
Schisandrin	*Staphylococcus aureus*; *Bacillus subtilis* [13]
Schisandrol A/B	*Escherichia coli*; *Pseudomonas aeruginosa* [14]
Gomisin A	*Streptococcus mutans*; *Enterococcus faecalis* [15]
Gomisin C	*Staphylococcus aureus* [16]
Deoxyschisandrin	*Vibrio parahaemolyticus* [17]
Tartaric Acid	*Streptococcus mutans* [18]
Triterpenoids	*Staphylococcus aureus*; *Bacillus subtilis* [19]
Essential Oils	*Staphylococcus aureus*; *Escherichia coli* [20,21]

**Table 2:** The primers for the genes of *inf B*, *tuf*, *sod A*, *ahp C* and *16S*.

Gene Name	Forward Primer (5′ → 3′)	Reverse Primer (5′ → 3′)
*16S*	GGCAAGCGTTATCCGGAATT	GTTTCCAATGACCCTCCACG
*inf B*	ACCAACTTCAAATCCTGATC	TGTAATTTCAACAGGCGTTG [27]
*tuf*	TCCTGGTTCAATTACACCACATACTG	GGAAATAGAATTGTGGACGATAGTTTGA [28]
*Sod A*	GCGTGTTCCCATACGTCTAAACC	TTGTGACTACACCAAACCAAGATAAT [29]
*ahp C*	CACGGCCAATTCCGTCA	TGACCCATCACAAACAATCACTC [30]

## Data Availability

The original contributions presented in this study are included in the article/Supplementary Materials. Further inquiries can be directed to the corresponding author.

## Supplementary Materials

The following supporting information can be downloaded at: https://www.mdpi.com/article/10.3390/vetsci13030285/s1, Table S1: The chemical components in Xiao Xue Teng; Figure S1: The chromatogram (A) and total ion chromatogram (B) of Xiao Xue Teng; Figure S2: The total ion chromatogram (TIC) of band A, band B, band C and band D; Figure S3: The densitometry of band A, band B, band C and band D analyzed using the Image J software. SDS-PAGE original image of total protein of *Staphylococcus aureus* ATCC 25923 treated with Xiao Xue Teng.
