# Supplementary material for: Bacteriostatic Mechanism of the Ethyl Acetate Extract from the Root of Schisandra propinqua (Wall.) Baill. var. sinensis Oliv (Xiao Xue Teng) Against Staphylococcus aureus
Source: Vet Sci. 2026 Mar 18;13(3):285. doi: 10.3390/vetsci13030285 (PMC13029955; doi:10.3390/vetsci13030285)
Supplement: Supplementary file 1 [file vetsci-13-00285-s001.zip › vetsci-4156489-supplementary.pdf]

Supplementary material: Bacteriostatic mechanism of the ethyl acetate extract from the root of *Schisandra propinqua* (Wall.)Baill.var. *sinensis* Oliv (Xiao Xue Teng) against *Staphylococcus aureus*

Lingyun Gu [1], Huifang Zhou [1], Qunxin Wang [1], Weidong Sun [1], Fuxin Chen [2], Tuo Li [3] and Chenghua He [1,*]

[1] College of Veterinary Medicine, Nanjing Agricultural University, Nanjing 210095, China; gulingyun@stu.njau.edu.cn (L.G.); 2024107089@stu.njau.edu.cn (H.Z.); 2024807144@stu.njau.edu.cn (Q.W.) swd100@njau.edu.cn (W.S.); hechenghua@njau.edu.cn (C.H.)
[2] School of Chemistry and Chemical Engineering, Xi'an University of Science and Technology, Xi'an 710054, China; chenfuxin@xust.edu.cn (F.C.);
[3] Dalian Institute of Marine Traditional Chinese Medicine, Dalian University, Dalian, Liaoning, China; lituo@dlu.edu.cn (T.L.)
* Correspondence: hechenghua@njau.edu.cn; Tel.: +86-025-8439-5227; Fax: +86-025-8439-8669

Contents

Table S1. S. The chemical components in the Xiao Xue Teng.

Figure S1. S. The chromatogram (A) and total ion chromatogram (B) of the Xiao Xue Teng.

Figure S2. S. The total ion chromatogram (TIC) of the band A, band B, band C and band D.

Figure S3. S. The densitometry of band A, band B, band C and band D analyzed by the Image J software. ** means *P*-value < 0.01.

Table S1. The chemical components in the Xiao Xue Teng.

Peak	Chemical name	Formula	Mass(Da)	Adduct	Extraction mass	Found mass	Error(ppm)	RT (min)
1	Schizanrin F	$C_{32}H_{34}O_{11}$	594.21011	^{+}H	595.21739	595.21774	0.6	18.93
2	E-Resveratrol trimethyl ether	$C_{17}H_{18}O_3$	270.12559	^{+}H	271.13287	271.13274	-0.5	20.02
3	Formononetin	$C_{16}H_{12}O_4$	268.07356	^{+}H	269.08084	269.08085	0.1	20.16
4	arisanlactone C	$C_{29}H_{40}O_{10}$	548.26215	^{+}H	549.26942	549.26809	-2.4	23.81
5	dehydrated schizandrin	$C_{24}H_{30}O_6$	414.20424	^{+}H	415.21152	415.21185	0.8	25.23
6	7-O-Methylcedrusin	$C_{20}H_{24}O_6$	360.15729	^{+}H	361.16457	361.16309	-4.1	27.63
7	benzoylgomisin Q	$C_{31}H_{36}O_9$	552.23593	^{+}H	553.24321	553.24202	-2.2	28.26
8	Manwuwezic Acid	$C_{30}H_{46}O_4$	470.33961	^{+}H	471.34689	471.34629	-1.3	28.86
9	tigloylgomisin P	$C_{28}H_{34}O_9$	514.22028	^{+}H	515.22756	515.2273	-0.5	29.01
10	Schisantherin A	$C_{30}H_{32}O_9$	536.20463	^{+}H	537.21191	537.21067	-2.3	29.03
11	alismol	$C_{15}H_{26}O$	222.19837	^{+}H	223.20564	223.20548	-0.7	29.16

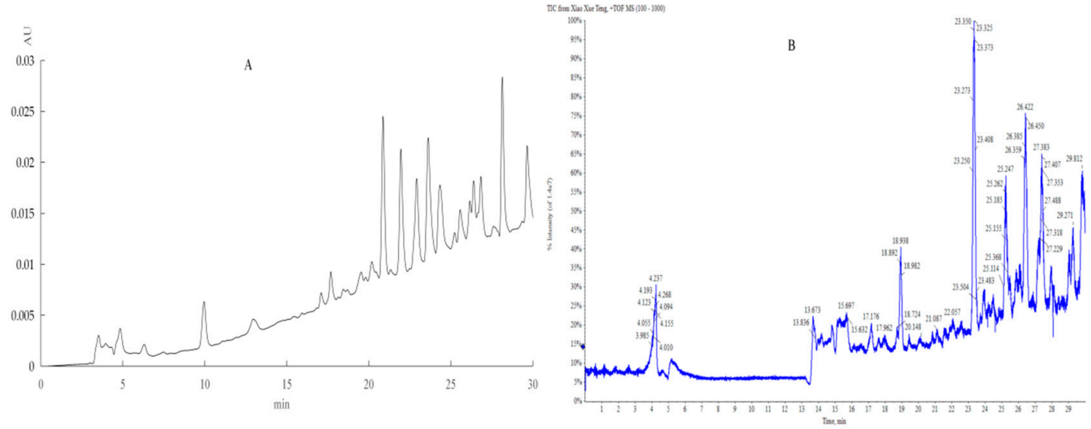

Figure S1. The chromatogram (A) and total ion chromatogram (B) of the Xiao Xue Teng.

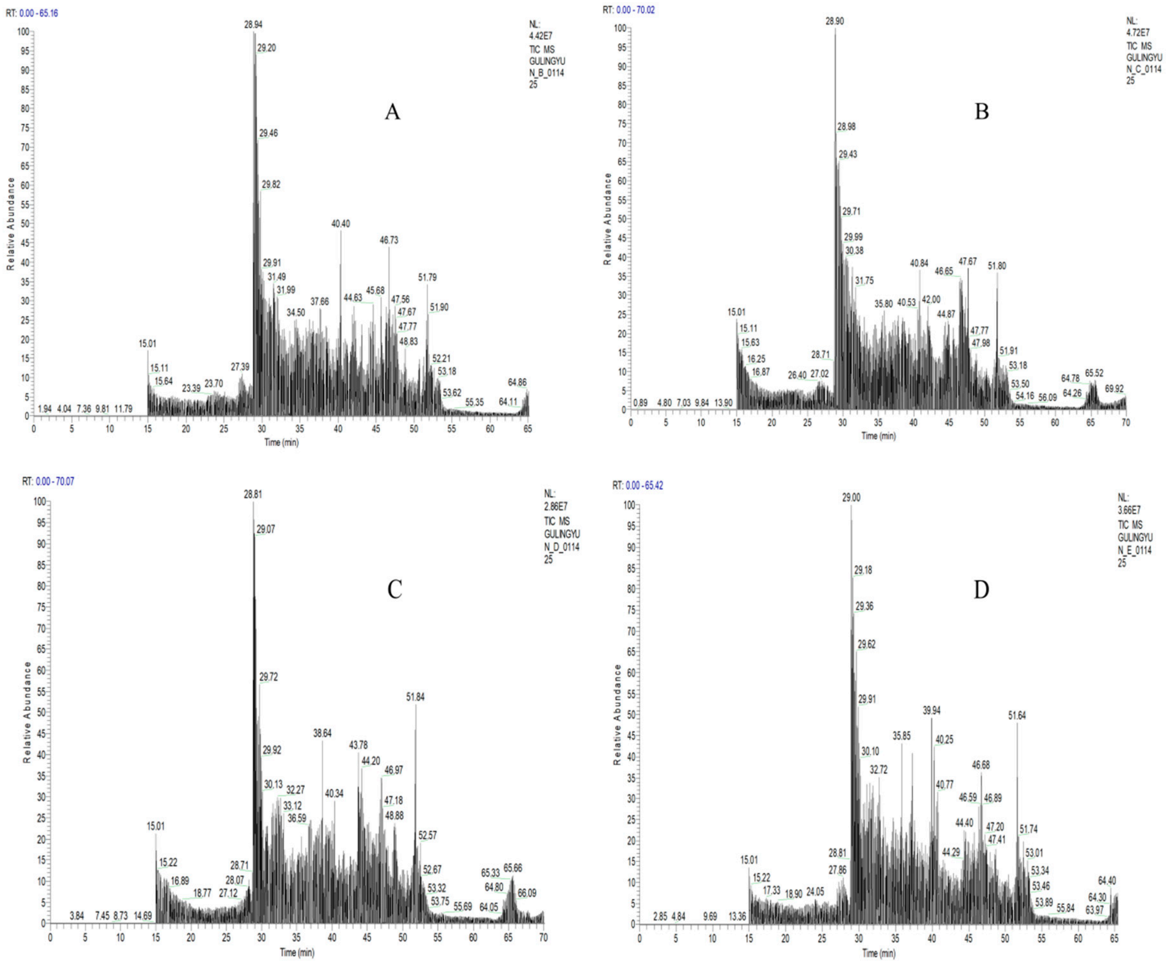

Figure S2. The total ion chromatogram (TIC) of the band A, band B, band C and band D.

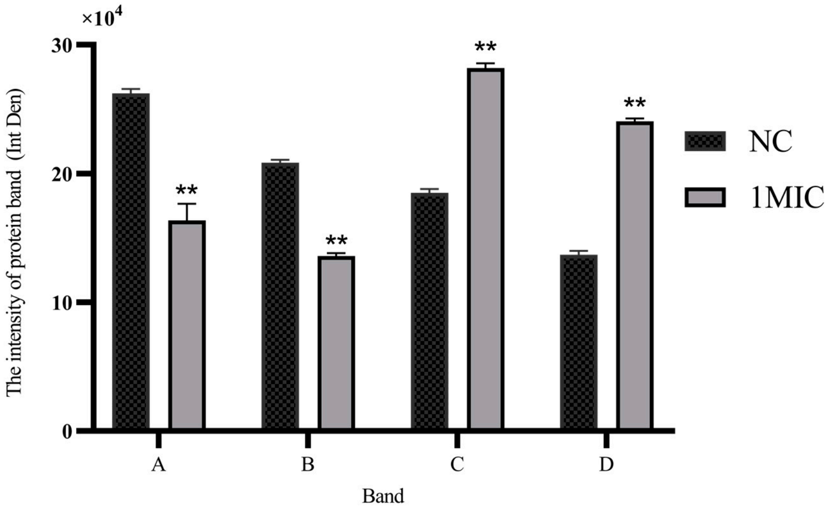

Figure S3. The densitometry of band A, band B, band C and band D analyzed by the Image J software. ** means P-value < 0.01.
