# Supplementary figures and images for: Bacteriostatic Mechanism of the Ethyl Acetate Extract from the Root of Schisandra propinqua (Wall.) Baill. var. sinensis Oliv (Xiao Xue Teng) Against Staphylococcus aureus
Source: Vet Sci. 2026 Mar 18;13(3):285. doi: 10.3390/vetsci13030285 (PMC13029955; doi:10.3390/vetsci13030285)

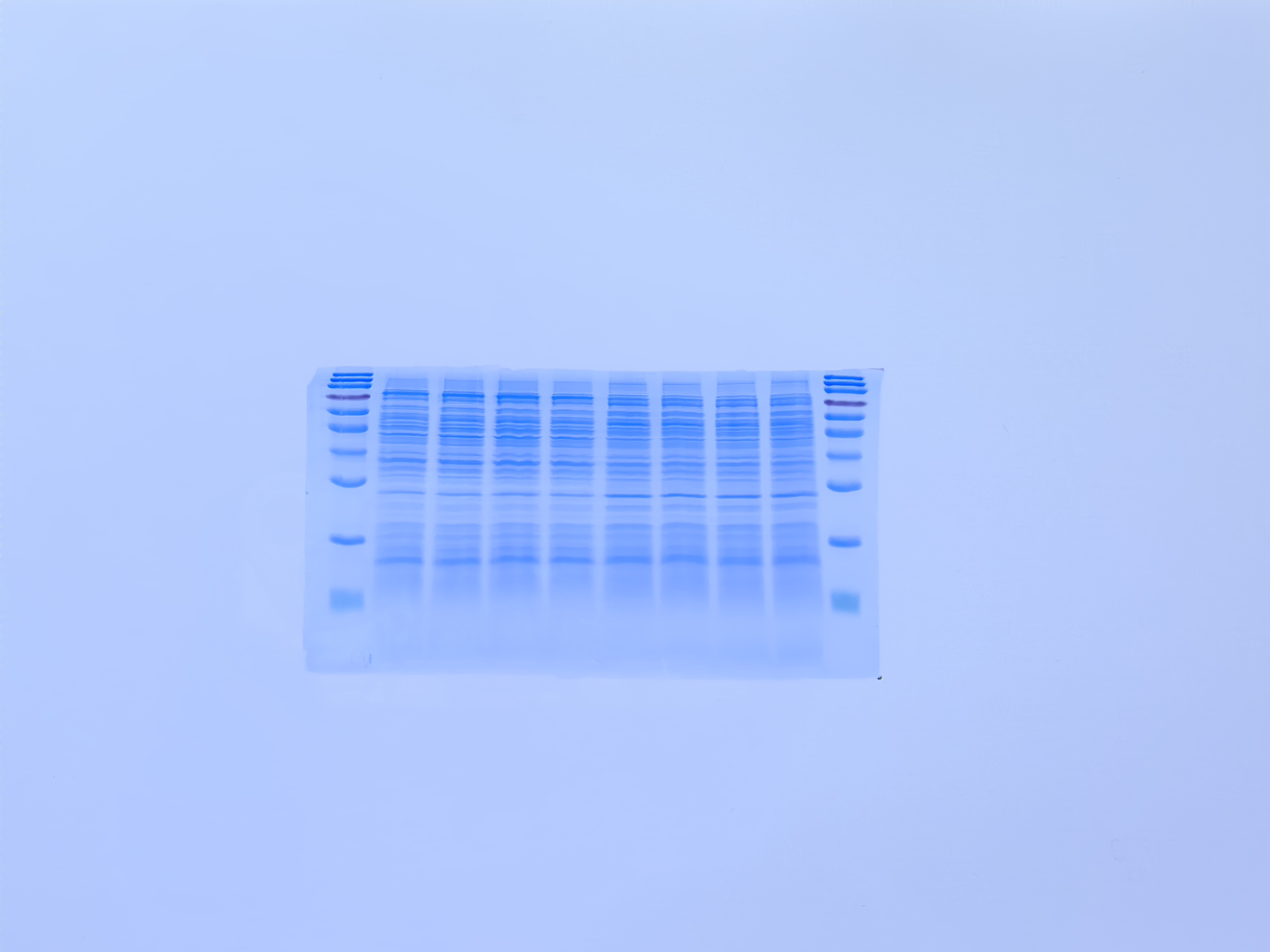

Supplement: Supplementary file 1 [file vetsci-13-00285-s001.zip › SDS-PAGE.jpg]
